# The Effect of Tuberculosis Treatment at Combination Antiretroviral Therapy Initiation on Subsequent Mortality: A Systematic Review and Meta-Analysis

**DOI:** 10.1371/journal.pone.0078073

**Published:** 2013-10-15

**Authors:** Heidi M. Soeters, Charles Poole, Monita R. Patel, Annelies Van Rie

**Affiliations:** Department of Epidemiology, Gillings School of Global Public Health, University of North Carolina at Chapel Hill, Chapel Hill, North Carolina, United States of America; Fundacion Huesped, Argentina

## Abstract

**Objective:**

We aimed to perform a systematic review and meta-analysis examining the impact of TB treatment at the time of combination antiretroviral therapy (cART) initiation on subsequent mortality.

**Methods:**

We searched PubMed, EMBASE, and selected conference proceedings for studies that report adult mortality on cART, stratified by TB treatment status at cART initiation. Stratified random-effects and meta-regression analyses were used to examine the influence of study and population characteristics.

**Results:**

22 eligible cohort studies reported data on 98,350 (range 74-15,225) adults, of whom 14,779 (15%) were receiving TB treatment at cART initiation. Studies of those receiving vs. not receiving TB treatment had an average mortality relative risk of 1.10 (95% confidence interval 0.87-1.40) at 1-3 months (based upon 8 estimates), 1.15 (0.94-1.41) at 6-12 months (11 estimates), and 1.33 (1.02-1.75) at 18-98 months (10 estimates) following cART initiation. However, there was a wide range of estimates and those at later time points were markedly heterogeneous. Meta-regression identified factors associated with elevated average risk estimates: lower median baseline CD4 counts and adjustment for baseline hemoglobin at 1-3 months; longer length of follow-up and women-only studies at 6-12 months; and not adjusting for BMI/weight at 18-98 months.

**Conclusions:**

Patients receiving TB treatment at cART initiation did not have a statistically significant estimated increase in short-term risk of all-cause mortality as compared to those not receiving TB treatment. TB treatment was significantly associated with increased mortality after about a year of cART, suggesting that patients with concurrent TB treatment at cART initiation may benefit from continued support after TB treatment completion.

## Introduction

Tuberculosis (TB) continues to threaten the health of people living with HIV (PLWH). Globally in 2011, 13% of incident TB cases were co-infected with HIV and an estimated 0.4 million TB deaths occurred among PLWH [1]. Access to combination antiretroviral therapy (cART) has dramatically increased survival, but a substantial number of PLWH die during the first year of cART. The majority of deaths occur within the first three months [2-5]. Autopsy studies have consistently shown TB to be an important cause of death in PLWH, both in the pre-cART [6-8] and cART eras [9]. 

In 2010, a meta-analysis of the effect of TB on mortality found PLWH also suffering from TB had a greater risk of mortality than TB-free individuals (hazard ratio [HR]: 1.8, 95% confidence interval [CI]: 1.4-2.3) [10]. Due to the paucity of studies reporting on patients on cART, the authors concluded that the effect of TB on mortality in PLWH exposed to cART must be further evaluated once more cohort study results become available.

Given the World Health Organization’s 2010 recommendation that all PLWH with TB be initiated on cART, regardless of CD4 count [11], and the goal of 100% cART coverage of co-infected patients by 2015 [12], many individuals will be initiating cART while concurrently on treatment for confirmed or clinically suspected active TB. PLWH who are also being treated for TB may experience a differential response to cART due to drug-drug interactions [13,14], an increased risk of drug toxicity [13,14], immune reconstitution inflammatory syndrome [15], and the potential for lower adherence due to the high pill burden [14]. The effect of TB treatment and its associated potential challenges and complications on a patient’s response to cART is not yet clear [16].

We aim to describe the impact of receiving TB treatment at the time of cART initiation on subsequent mortality among HIV-infected adults. We performed separate analyses to identify the effect at 1-3 months, 6-12 months, and 18-98 months.

## Methods

### Search strategy and selection criteria

To investigate the effect of TB treatment at the time of cART initiation on mortality, we carried out a systematic and sensitive search using an *a priori* protocol developed according to PRISMA guidelines [17] (Checklist S1). We searched PubMed and EMBASE, as well as abstract databases from the 2009 to 2012 Conferences on Retroviruses and Opportunistic Infections, International Union Against Tuberculosis and Lung Disease World Conferences on Lung Health, and International AIDS Society conferences. The search terms “HIV AND Tuberculosis AND (Viral Load OR CD4 lymphocyte count OR Mortality) AND Antiretroviral therapy” were used to identify relevant articles in PubMed and EMBASE. Searches were performed on March 1, 2013 and included original human subjects cohort studies published since 1997 (the start of the cART era). Additional articles were identified from reference lists, reviews, and Web of Science citation lists.

H.M.S. and A.V.R. independently reviewed titles and abstracts of original studies retrieved by the search. H.M.S. reviewed full-text and references of selected articles. H.M.S. and M.R.P. independently abstracted study data from full reports; discrepancies were resolved by consensus or consultation with A.V.R. and C.P.

Studies were included if they included both antiretroviral-naïve HIV-infected individuals receiving and not receiving TB treatment at cART initiation, and reported mortality after cART initiation stratified by TB treatment status at cART initiation. cART was defined as a treatment regimen containing three or more antiretrovirals. Though we sought reports of cART-naïve patients, studies with ≤10% antiretroviral-experienced patients or patients only previously exposed to a single intrapartum dose of nevirapine were also included. Studies of children <14 years of age were excluded. No additional exclusion criteria or language restrictions were imposed.

### Data extraction

The following information, if available, was abstracted from each article: first author surname; publication year; study design; study dates; length of follow-up; geographic location; clinical setting; sample size; number receiving and not receiving TB treatment; if TB treatment was the main exposure of interest; types of TB included; culture confirmation of TB cases; TB site; timing of TB treatment in relation to cART initiation; percentage male; mean or median participant age; proportion treatment-naïve; cART regimen; baseline median CD4 count and HIV-RNA; measure of effect or event counts; covariate adjustment; proportion lost-to-follow-up; and if mortality was confirmed using a national death registry. 

### Statistical analysis

Reported mortality effect-measure estimates over any length of time were abstracted. If only survival proportions among those receiving or not receiving TB treatment were reported, a risk ratio (RR) and 95% CI were calculated, as the RR approximates the HR for an uncommon outcome. If an effect-measure estimate was reported for those not receiving vs. receiving TB treatment, the inverse of the reported effect-measure estimate and CI were included. If only a p-value from a univariate logistic regression model was presented, the 2x2 table was reconstructed and a RR was calculated. Standard error estimates were inferred from reported CIs by [ln(upper limit) - ln(lower limit)]/3.92 [18].

As tuberculosis is successfully treated in most patients after six months of treatment, and the hazard of mortality following cART initiation is not constant, we grouped available cumulative effect estimates according to length of follow-up: 1-3 months, 6-12 months, and 18-98 months. None of the eligible studies reported estimates at 4-5 months or 13-17 months following cART initiation. If a study reported multiple estimates within a time category, the time frame closest to the category midpoint was included in the primary analysis and other time estimates were examined in sensitivity analyses.

The method of moments estimate of the among-populations variance (τ^2^) and random-effects summarization using unconditional variances were used to combine relative risks in the three groups [19]. The p-values for a standard chi-square homogeneity test statistic were used to assess overall consistency among the effect estimates. τ^2^ was used to calculate 95% population effects intervals [20] (where 95% of populations are estimated to have their relative risks), opposite effects proportions [21] (proportion of populations estimated to experience a relative risk on the opposite side of the estimated mean, in this case below unity), and 95% prediction intervals [21] (95% of these intervals will cover the true value estimated by a future study). Stratified and random-effects meta-regression analyses were used to calculate stratum-specific summary measures and 95% CIs, along with ratios of the average RRs as described by Thompson and Sharp [22]. Study characteristics with at least two studies per stratum were eligible for inclusion in the meta-regression.

Funnel plots of ln(mortality relative risk) vs. the inverse-variance weight of studies for each time category were visually examined for asymmetry and statistically assessed using methods proposed by Begg [23] and Egger [24] and the trim-and-fill method of Duval and Tweedie [25]. STATA (version 12, Stata corporation, College Station, TX) was used for these analyses.

## Results

### Selected studies

**Figure 1:**
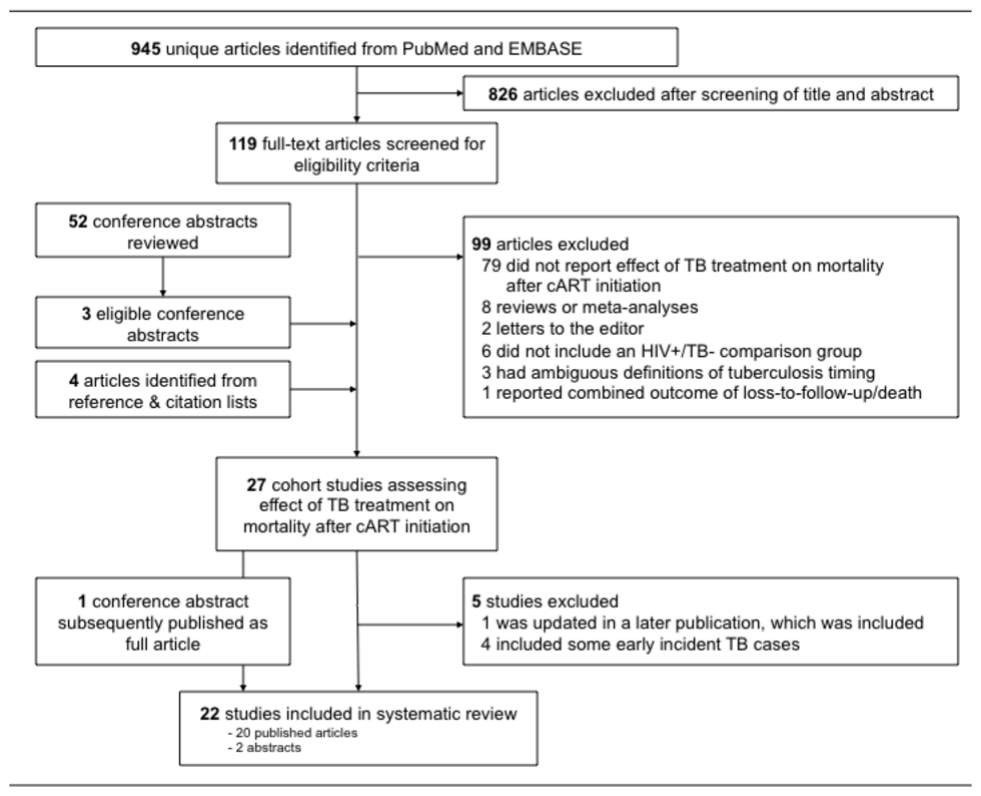
Identification and selection of eligible studies.

997 unique abstracts were reviewed: 824 from PubMed, 121 additionally from EMBASE, and 52 from conference proceedings (Figure 1). Of these, 119 full-text articles were selected for review. In total, 20 articles [2,26-44] and three conference abstracts [45-47] were eligible. Four additional articles met our inclusion criteria: two from reference lists [48,49] and two from Web of Science citation searches [50,51]. One included abstract [46] was subsequently published as a full article [52]; only data from the full article was included. Five eligible studies were excluded: one [43] because a 2012 paper [29] provided an updated estimate; four because they included some early incident TB cases in their TB treatment-exposed group [28,38,39,42].

### Study and population characteristics

The 22 final studies provided data on 98,350 PLWH, of which 14,779 (15%) were receiving TB treatment at cART initiation. Selected study and population characteristics are displayed in Table 1. All were cohort studies. Most publications assessed mortality after cART initiation, regardless of regimen type, though some reported estimates specific to nevirapine-[31,41,48,49] or efavirenz-based [26,31,37] therapy. See Table S1 for cART regimens used by each study. All studies used cART initiation as the time origin, except one which began at the commencement of cART education and adherence sessions, with most patients starting cART a month or two later [52]. Four studies included women previously exposed to a single intrapartum dose of nevirapine [30-33], and two studies included 3% [29] and 6% [36] antiretroviral-experienced patients.

**Table 1:** Characteristics of 22 studies reporting the effect of TB treatment on mortality after cART initiation among HIV-infected adults.

**Study**	**Publication year**	**Geographic location**	**Sample size**	**TB treatment, N (%)**		**Main exposure is TB**	**Study design**	**cART regimen** ^a^	**Naïve**, **%**	**Male, %**	**Mean age, years**	**Median baseline CD4 count** ^b^ **, cells/μL**	**Median baseline HIV-RNA, log_10_ copies/mL**	**Lost to follow-up, %**	**Overall mortality** ^c^,**%**	**Confirmed mortality with national death registry**	**Duration of follow-up, months**
Bassett[52]	2012	South Africa	951	343	(36)	Yes	P	All	100	41	36	90	NS	7	10	No	12
Bera[30]	2009	South Africa	385	25	(7)	No	P	All	100	0	28	173	4.6	4	2	No	8
Bhowmik[27]	2012	India	743	285	(38)	No	R	All	100	66	35	140	NS	NS	7	No	12
Boulle (a)[31]^d^	2008	South Africa	1935	209	(11)	Yes	P	NVP	100	21	31	110	5	6	4	No	6
Boulle (b)[31]^d^	2008	South Africa	2035	1074	(53)	Yes	P	EFV	100	40	33	78	5.2	6	6	No	6
Boulle (a,b)[32] ^e^	2010	South Africa	7323	2760	(38)	No	P	All	100	32	33	101	5.1	10	NS; 16	Yes	3; 3-60
Chu[47]	2011	Uganda	15225	1177	(8)	Yes	P	All	100	NS	NS	NS	NS	NS	7	No	65
Dao[33]	2011	Zambia/Kenya	661	56	(8)	No	P	All	100	0	32	147	5	5	8	No	12
DeSilva[34]	2009	Nigeria	1552	251	(16)	No	R	All	100	29	34	112	NS	9	7	No	24
Dronda[35]	2011	Spain	1986	110	(6)	Yes	P	All	100	76	38	196	5.0	7	3	No	47
Greig[36]	2012	Sub-Saharan Africa	14523	1159	(8)	No	R	All	94	35	36	133	NS	12	7	No	30
Gupta[51]	2013	South Africa	1544	464	(30)	Yes	P	All	100	30	34	98	4.9	21	13	No	100
Lartey[37]	2011	Ghana	74	34	(46)	Yes	P	EFV	100	49	NS	83	5.4	11	9	No	11
Liechty[40]	2007	Uganda	377	32	(8)	No	P	All	100	29	38	50	5.5	0	6	No	3
Makombe (a,b) [49]^e^	2007	Malawi	12485	1339	(11)	Yes	R	NVP	100	NS	NS	NS	NS	11	12; 13	No	6; 12
Manosuthi[41]	2010	Thailand	140	70	(50)	Yes	P	NVP	100	68	36	31	5.6	11	6	No	48
Mugusi (a,b)[26]^e^	2012	Tanzania	449	194	(43)	Yes	P	EFV	100	42	40	92	5.7	12	3; 11	No	1; 11
Mutevedzi (a,b)[50]^f^	2011	South Africa	7927	1752	(22)	No	R	All	100	33	34	117	4.4	11	5; 3	No	3; 3-12
Mutevedzi (c,d)[50]^f^	2011	South Africa	919	175	(19)	No	R	All	100	44	54	127	4.5	11	6; 6	No	3; 3-12
Nguyen[45]	2011	Vietnam	370	NS	NS	No	R	All	100	66	33	NS	NS	NS	31	No	60
Stringer (a,b)[2]^e^	2006	Zambia	14306	1562	(11)	No	P	All	100	39	35	143	NS	21	6; 9	No	3; 18
Westreich (a,b)[29]^e^	2012	South Africa	7512	1197	(16)	Yes	P	All	97	34	35	88	NS	NS	7; 9	Yes	36; 54
Zachariah[44]	2006	Malawi	1507	225	(15)	No	P	All	100	34	35	123	NS	3	8	No	3
Zachariah[48]	2009	Malawi	2289	196	(9)	No	R	NVP	100	31	35	NS	NS	5	9	No	3

Abbreviations: cART, combination antiretroviral therapy; EFV, Efavirenz-based cART; HIV, human immunodeficiency virus; NS, not specified; NVP, Nevirapine-based cART; P, prospective study; R, retrospective study; TB, tuberculosis.

a See Table S1 for detailed information on cART regimens from each study, if available

b See Table S4 for baseline CD4 cell count stratified by TB treatment status from each study, if available

c If a study reported relative risks for multiple time periods, an overall mortality estimate is indicated for each respective time period

d (a) Nevirapine-based cART; (b) Efavirenz-based cART

e For these studies, the letters after the author’s name refer to the estimates reported at differing time points. For example, Makombe (a) refers to the 6-month estimate and Makombe (b) refers to the 12-month estimate.

f (a,b) patients <50 years old; (c,d) patients ≥50 years old

Ten studies examined TB treatment at cART initiation as the main exposure of interest [26,29,31,35,37,41,47,49,51,52]. The other twelve studies examined TB treatment as a secondary exposure; five examined any predictors of mortality [27,33,34,44,48], three aimed to describe general cART outcomes [2,30,32], and one each examined the primary exposures of integrated vs. vertical HIV programs [36], positive serum cryptococcal antigen [40], age [50], and hepatitis B and C co-infections [45]. The type of TB being treated varied across studies (Table S2). Only two studies had a substantial subset of bacteriologically-confirmed TB cases [51,52] while others included both confirmed and probable TB cases. Most articles included any TB being treated at the time of cART initiation, whereas others used a specific time period such as new TB diagnosis at study entry [26,38] or diagnosis ≥1 month prior to enrollment [41]. Nine studies reported detail on the duration of TB treatment at the time of cART initiation (Table S3). One study focused solely on pulmonary TB [29], and one censored patients in the reference group that developed incident TB [31]. 

Mortality was mainly assessed using medical records and/or confirmation from family or health workers, however two studies additionally used national death registries [29,32]. In an effort to quantify late mortality, two studies excluded deaths occurring in the first three months [32,50]. Overall loss-to-follow-up was reported by 19 studies and ranged from 0% to 21% (median 9%).

Two studies were limited to women only [30,33]. Mean patient age ranged from 28 to 40 years (median 35 years), with the youngest included age being 14 years [31]. One study stratified mortality estimates according to age [50]. Median baseline HIV-RNA ranged from 4.4 to 5.7 log_10_ copies/mL (median 5.1), and baseline CD4 count ranged from 31 to 196 cells/μL (median 111). See Table S4 for baseline CD4 count stratified by TB treatment status from each study, if available.

### Relative risk of mortality

**Figure 2:**
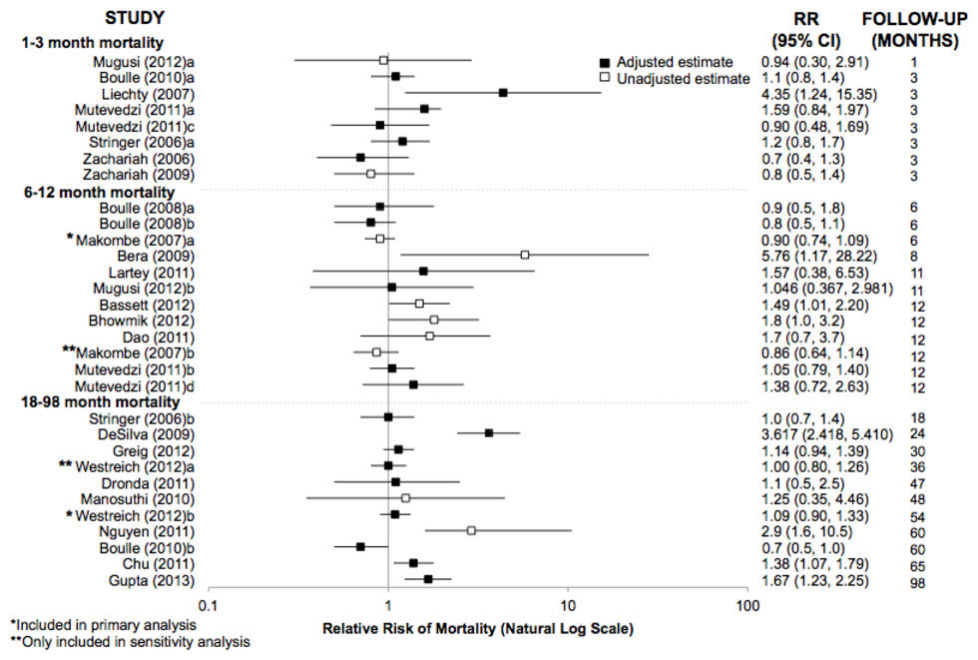
Forest plot of mortality relative risks reported by 22 studies. The relative risks correspond to the estimated effect of receiving vs. not receiving TB treatment at the time of cART initiation on subsequent mortality among HIV-infected adults. Estimates are ordered according to length of follow-up time. Estimates were abstracted according to the precision used by the original authors; estimates calculated using available data are reported to 2 decimal places. Abbreviations: cART, combination antiretroviral therapy; CI, confidence interval; HIV, human immunodeficiency virus; RR, relative risk; TB, tuberculosis.

Thirty-one cumulative mortality relative risks were reported or calculated among those receiving vs. not receiving TB treatment at cART initiation (Figure 2). Most were HRs, though eight RRs [26,30,37,41,47,49,52], four odds ratios [27,40,44,48], and one incidence rate ratio [51] were also included. Among the 21 adjusted estimates, 20 adjusted for baseline CD4 count, 19 for gender, 18 for age, 13 for BMI or weight, eight for hemoglobin, and two for adherence (defined as timeliness of pharmacy attendance). Including only the estimate corresponding to the longest follow-up time from each study, the summarized relative risk of mortality in those receiving vs. not receiving TB treatment across all time periods was 1.24 (95% CI 1.05-1.46, τ^2^ = 0.097).

Eight relative risks of mortality by 1-3 months were reported, without evidence of substantial heterogeneity (homogeneity p-value = 0.11, Table 2). The random-effects summary estimate was 1.10 (95% CI 0.87-1.40, τ^2^ = 0.045). The eleven mortality relative risks by 6-12 months produced a random-effects summary estimate of 1.15 (95% CI 0.94-1.41, τ^2^ = 0.041). By 18-98 months, the summarized relative risk indicated increased mortality among patients receiving TB treatment (random effects relative risk [RR_RE_] = 1.33, 95% CI 1.02-1.75, τ^2^ = 0.134), based upon 10 estimates. There was evidence of considerable heterogeneity for the 6-12 and 18-98 month estimates (homogeneity p-values 0.06 and <0.01, respectively).

**Table 2:** Meta-analysis results for the effect of TB treatment on mortality, by length of follow-up time.

**Length of follow-up time**	**1-3 months**	**6-12 months**	**18-98 months**
No. of studies	8	11	10
Homogeneity p-value	0.106	0.063	<0.001
Estimate of between-study variance (τ^2^)	0.045	0.041	0.134
RR_RE_ (95% CI)	1.10 (0.87, 1.40)	1.15 (0.94 1.41)	1.33 (1.02, 1.75)
95% population effects interval	(0.73, 1.67)	(0.77, 1.71)	(0.65, 2.73)
Opposite effects proportion	32.2%	24.8%	21.6%
95% prediction interval	(0.62, 1.97)	(0.69, 1.91)	(0.55, 3.23)

Abbreviations: CI, confidence interval; RR_RE_, random-effects summary relative risk; TB, tuberculosisRR: risk ratio

### Meta-regression

Meta-regression results are displayed in Table S5. For 1-3 month mortality, lower median baseline CD4 counts and adjustment for baseline hemoglobin appear to produce somewhat higher relative risks. Among the 6-12 month estimates, length of follow-up appeared to have the most influence and produced the most homogenous strata, suggesting that this 6-month window may be too wide. At 6 to 9 months following cART initiation, those receiving vs. not receiving TB treatment had a RR_RE_ of 0.93 (95% CI 0.68-1.28, homogeneity p-value = 0.14), whereas at 11 to 12 months the RR_RE_ was 1.29 (95% CI 1.06-1.56, homogeneity p-value = 0.61), indicating an increased risk of mortality after completion of TB treatment. Studies limited to women only also reported higher RRs (RR_RE_ = 2.57, 95% CI 0.83-7.98). Among 18-98 month estimates, a longer follow-up time does not appear to influence the results. However, adjustment for BMI or weight tended to move the relative risk toward the null (RR_RE_ = 1.01, 95% CI 0.84-1.21).

### Funnel plot analysis

For 1-3 month estimates, the funnel plot gives the visual appearance of a slight skew to the left (Document S1), but both tests for asymmetry yielded p-values of 0.8, and only one hypothetical missing result was imputed on the right by trim-and-fill analysis, with little influence on the summary results. In contrast, funnel plots for 6-12 and 18-98 months were skewed more noticeably to the right and the tests for asymmetry yielded lower p-values (Begg p = 0.2, Egger p = 0.03 for 6-12 months, Begg p = 0.3 and Egger p = 0.4 for 18-98 months). Trim-and-fill imputed four hypothetically missing results at 6-12 months and two at 18-98 months, all on the left. The imputation shifted the estimate of the random-effects average RR from 1.15 to 1.02 at 6-12 months and from 1.33 to 1.14 at 18-98 months.

### Sensitivity analyses

As Makombe et al. (2007) [49] contained multiple estimates of mortality in the 6-12 month time frame, only the six-month estimate was included in primary analyses. The analysis was then repeated, substituting in the 12-month estimate. As this alternative estimate was similar in point estimate and precision to the 6-month estimate, the substitution did not affect the results or our conclusions. Likewise, Westreich et al. (2012) [29] reported estimates at 36 months and 54 months. Regardless of which was included, the analysis results did not substantially differ. Additionally, as some studies reported asymmetrical confidence intervals, a sensitivity analyses assessed their impact on the summary measures (Document S2).

## Discussion

This systematic review and meta-analysis of studies reporting the effect of TB treatment at cART initiation on subsequent mortality among PLWH initiating therapy found that TB treatment did not significant affect mortality on cART in the short-term, but was associated with increased mortality after about a year of cART. These results are important given the many concerns about co-treatment including drug-drug interactions, overlapping drug toxicities, immune reconstitution inflammatory syndrome, and high pill burden. Our findings were not sensitive to geographical study location (Africa vs. Asia or Europe).

Overall, the summary estimates for early mortality were lower than estimates for later mortality. This finding may be contrary to expectations, but could be due to several factors. First, patients on concurrent TB therapy at cART initiation may experience a lower short-term risk of all-cause mortality because TB medications are also effective against infectious diseases other than TB [53]. Second, most PLWH receiving TB treatment often receive co-trimoxazole preventive therapy, which further reduces the risk of death from non-TB infectious diseases. Third, prior to cART initiation, PLWH on TB treatment may have been engaged in care for longer than other PLWH. Fourth, TB deaths can occur early during TB treatment, i.e., prior to the initiation of cART, creating possible left-censoring. However, PLWH not diagnosed with TB also may die prior to cART initiation, and studies did not provide enough information to determine if left-censoring was differential between the two groups. Fifth, undiagnosed and untreated TB among the comparison group may have biased estimates of early mortality toward the null. Autopsy studies consistently show that undiagnosed TB continues to be a major cause of death among HIV-infected adults [6-8], even in the cART era [9]. 

This meta-analysis has a number of limitations. Since being treated for active TB is not an exposure suitable for a randomized controlled trial, all studies included in our meta-analysis were observational and subject to biases. First, the outcome of mortality may have been misclassified in people lost-to-follow-up. Rates of follow-up loss were variable in the included studies, ranging from 0% to 21%. cART programs with high losses of patients and incomplete death ascertainment can seriously underestimate mortality, with 12% to 87% of patients loss-to-follow-up in fact being deceased [54]. Misclassification of deaths would only produce bias in the estimated relative risks if this misclassification was differential between compared groups [55] or dependent on errors in measuring other variables. This is especially relevant for mortality estimates during the first six months of cART, when loss-to-follow-up may be differential due to regular follow-up for TB treatment. We attempted to examine if mortality confirmation by national death registry affected mortality effect estimates. This was only possible for two studies, both with 18-98 months of follow-up. 

Second, the exposure, prevalent TB treatment at cART initiation, captures both confirmed active TB disease and exposure to anti-tuberculosis drugs for clinically suspected TB. This combined exposure is useful from a health systems perspective, particularly in low-resource countries, where active TB cannot always be confirmed, especially in PLWH. The included studies used a variety of methods for determining who had active TB and should receive treatment, and no studies were exclusively limited to bacteriologically-confirmed TB cases. Consequently, active TB could have been misclassified and some patients included in this meta-analysis may have received TB treatment even though they did not have TB. In addition, some patients with active TB may not have been diagnosed. 

Third, there was much heterogeneity in the duration of TB treatment prior to cART initiation, with some patients on TB therapy for six months and others beginning TB treatment and cART concurrently. While the timing of TB treatment in relation to cART initiation is an important variable to consider when evaluating mortality [43,56-59], the included studies did not provide enough information on duration of TB treatment to systematically evaluate its effect on our results. Future studies should describe the duration of TB treatment in more detail to facilitate meta-analysis, though a pooled patient-level analysis or randomized controlled trial are study designs better suited to assess this.

Some degree of funnel plot asymmetry was apparent for each time period, possibly due to publication bias or other factors. A direct effect measure was not available from each study, and published estimation methods [18] were used to calculate the effect measure for five studies. These estimation techniques involve a number of assumptions and may have introduced bias or affected variance estimates. Additionally, in 12 of 22 studies, TB treatment was not the primary exposure and covariates included in multivariate models may differ from ideal confounder adjustment for this research question. Some studies only included TB treatment in their multivariable model for mortality because it was a statistically significant predictor, which may explain some funnel plot asymmetry. 

It was difficult to examine the influence of study or population characteristics on the effect estimates, as grouping studies by specific characteristics produced small strata with imprecise estimates. Furthermore, some characteristics are correlated. For example, risk ratios and odds ratios tended to be higher than hazard ratios, but the former also tended to be unadjusted estimates. Also, as studies adjusted for sets of key covariates, it was difficult or impossible to separate out the influence of adjusting for a specific covariate (see footnotes of Table S5).

Given the patient characteristics of the included studies, the results are most generalizable to therapy-naïve adults in Sub-Saharan Africa with relatively low CD4 counts at cART initiation. More studies in populations outside of sub-Saharan Africa, particularly in North America, would be useful additions to the literature. This review only includes studies among adults; it is unknown whether the relationship holds in children. Similarly, it is possible that cART regimen would modify the effect of TB treatment due to drug-drug interactions, and regimen-specific estimates would be of most use to clinicians treating co-infected patients. 

In conclusion, our results reinforce the concept [29] that TB treatment does not increase early mortality after cART initiation — the issue is undiagnosed and untreated TB — underscoring the need for intensified case finding to reduce early mortality associated with undiagnosed TB in PLWH. After about a year of cART, TB treatment was associated with increased mortality, despite the possibility of downward biases. This association should be elucidated in future studies, with an emphasis on separating possible effects of TB treatment from effects of active TB itself. In the meantime, patients receiving concurrent TB treatment at cART initiation may benefit from continued support after TB treatment completion.

## Supporting Information

Table S1 **Combination antiretroviral therapy regimens utilized in each study.**
(PDF)

Table S2 **Types of TB included, by study.**
(PDF)

Table S3 **Timing of TB treatment in relation to cART initiation, by study.**
(PDF)

Table S4 **Median (IQR) baseline CD4 cell count by TB treatment status, if available.**
(PDF)

Table S5 **Meta-regression results for the effect of TB treatment on mortality.**
(PDF)

Document S1 **Funnel plots of mortality relative risks and inverse-variance weights.**
(PDF)

Document S2 **Sensitivity analysis of asymmetrical confidence intervals.**
(PDF)

Checklist S1 **PRISMA checklist.**
(PDF)
