# Supplementary material for: The Effect of Tuberculosis Treatment at Combination Antiretroviral Therapy Initiation on Subsequent Mortality: A Systematic Review and Meta-Analysis
Source: PLoS One. 2013 Oct 15;8(10):e78073. doi: 10.1371/journal.pone.0078073 (PMC3797056; doi:10.1371/journal.pone.0078073)
Supplement: Table S1 — Combination antiretroviral therapy regimens utilized in each study. (PDF) [file pone.0078073.s001.pdf]

Table S1. Combination antiretroviral therapy regimens utilized in each study

Study	cART regimen(s)
Bassett 2012	"standard ART regimens as per contemporaneous South African guidelines"
Bera 2009	96.4% EFV-based cART<br>4.2% NVP-based cART<br>2.9% ZDV-based cART<br>97.4% d4T-based cART
Bhowmik 2012	First-line: EFV/NVP + ZDV/d4T + 3TC
Boulle 2008 (a)	NVP + 2 NRTIs
Boulle 2008 (b)	EFV + 2 NRTIs
Boulle 2010 (a,b)	8.8% EFV + ZDV + 3TC<br>8.4% NVP + ZDV + 3TC<br>43.6% EFV + d4T + 3TC<br>38.5% NVP + d4T + 3TC<br>0.8% other
Chu 2011	Not specified
Dao 2011	First-line: EFV/NVP + ZDV/d4T + 3TC
DeSilva 2009	Most patients on first-line regimens (most common: NVP + d4T + 3TC)<br>10 patients received combined lopinavir/ritonavir as part of their regimen
Dronda 2011	Not specified
Gupta 2013	"predominantly EFV-based cART"
Greig 2012	Not specified
Lartey 2011	600 mg EFV + 400/300mg didanosine + 300 mg 3TC once daily
Liechty 2007	First-line: EFV/NVP + d4T + 3TC
Makombe 2007 (a,b)	First-line: NVP + d4T + 3TC
Manosuthi 2010	NVP + d4T + 3TC<br>d4T switched to tenofovir or ZDV if d4T-related adverse events developed
Mugusi 2012 (a,b)	With prevalent TB: 50.7% EFV + d4T + 3TC, 49.3% EFV + ZDV + 3TC<br>Without prevalent TB: 16.9% EFV + d4T + 3TC, 83.1% EFV + ZDV + 3TC
Mutevedzi 2011 (a,b,c,d)	First-line: EFV/NVP + d4T + 3TC
Nguyen 2011	Not specified
Stringer 2006 (a,b)	First-line: NVP + ZDV/d4T + 3TC<br>In patients receiving acute-phase TB therapy with CD4 cell count <50 cells/μL: EFV-based cART
Westreich 2012 (a,b)	With prevalent TB: 95.2% EFV-based cART, 3.0% NVP-based cART, 1.8% other<br>Without prevalent TB: 79.5% EFV-based cART, 9.5% NVP-based cART, 11.1% other
Zachariah 2006	93.7% fixed dose NVP + d4T + 3TC<br>4.4% EFV + d4T + 3TC<br>1.3% NVP + ZDV + 3TC<br>0.3% EFV + ZDV + 3TC<br>0.3% ZDV + didanosine + nelfinavir
Zachariah 2009	99.8% NVP + d4T + 3TC<br>0.2% NVP + ZDV + 3TC

Abbreviations: 3TC, lamivudine; ART, antiretroviral therapy; cART, combination antiretroviral therapy; d4T, stavudine; EFV, efavirenz; NRTI, nucleos(t)ide reverse-transcriptase inhibitor; NVP, nevirapine; TB, tuberculosis; ZDV, zidovudine
