# Supplementary material for: The Effect of Tuberculosis Treatment at Combination Antiretroviral Therapy Initiation on Subsequent Mortality: A Systematic Review and Meta-Analysis
Source: PLoS One. 2013 Oct 15;8(10):e78073. doi: 10.1371/journal.pone.0078073 (PMC3797056; doi:10.1371/journal.pone.0078073)
Supplement: Table S2 — Types of TB included, by study. (PDF) [file pone.0078073.s002.pdf]

Table S2. Types of TB included, by study

Study	Types of TB included
Bassett 2012	Newly diagnosed by sputum culture at cART enrollment or previously diagnosed and currently on treatment
Bera 2009	Concurrently on TB treatment at cART initiation
Bhowmik 2012	Concurrently on TB treatment at cART initiation
Boulle 2008 (a)	Concurrent TB treatment at cART initiation and for ≥14 days post cART initiation. TB diagnosed by microscopy, culture or clinical algorithm.
Boulle 2008 (b)	Concurrent TB treatment at cART initiation and for ≥14 days post cART initiation. TB diagnosed by microscopy, culture or clinical algorithm.
Boulle 2010 (a,b)	On TB treatment at cART initiation
Chu 2011	Concurrent active TB diagnosis at cART initiation
Dao 2011	Concurrent active TB diagnosis at cART initiation
DeSilva 2009	Concurrent TB diagnosis and treatment at cART initiation. TB co-infection assessed using patient files and pharmacy records.
Dronda 2011	Definite or presumptive diagnosis of TB in 6 months prior to cART initiation
Greig 2012	Current TB diagnosis at cART initiation
Gupta 2013	On TB treatment at cART initiation
Lartey 2011	On TB treatment at cART initiation
Liechty 2007	Concurrent active TB diagnosis at cART initiation
Makombe 2007 (a,b)	Concurrent active TB diagnosis at cART initiation
Manosuthi 2010	Receiving rifampicin for active TB ≥1 month prior to cART enrollment
Mugusi 2012 (a,b)	Newly diagnosed at cART enrollment by smear microscopy, histology or clinical criteria
Mutevedzi 2011 (a,b,c,d)	Concurrent TB treatment at cART initiation
Nguyen 2011	Active TB at cART initiation
Stringer 2006 (a,b)	Active TB at cART initiation
Westreich 2012 (a,b)	On treatment for pulmonary TB at cART initiation
Zachariah 2006	On treatment for active TB at cART initiation
Zachariah 2009	Active TB at cART initiation

Abbreviations: cART, combination antiretroviral therapy; TB, tuberculosis
