# Supplementary material for: The Effect of Tuberculosis Treatment at Combination Antiretroviral Therapy Initiation on Subsequent Mortality: A Systematic Review and Meta-Analysis
Source: PLoS One. 2013 Oct 15;8(10):e78073. doi: 10.1371/journal.pone.0078073 (PMC3797056; doi:10.1371/journal.pone.0078073)
Supplement: Table S3 — Timing of TB treatment in relation to cART initiation, by study. (PDF) [file pone.0078073.s003.pdf]

Table S3. Timing of TB treatment in relation to cART initiation, by study

Study	Timing of TB treatment in relation to cART initiation
Bassett 2012	144 newly diagnosed by sputum culture at cART enrollment<br>199 previously diagnosed and on TB treatment at cART enrollment
Bera 2009	Not specified
Bhowmik 2012	Not specified
Boulle 2008 (a)	Patients only included as prevalent TB in the analysis if they continued TB treatment past 14 days post cART initiation.<br>Duration of TB treatment at cART initiation [median (IQR)]: 87 (60-135) days
Boulle 2008 (b)	Patients only included as prevalent TB in the analysis if they continued TB treatment past 14 days post cART initiation.<br>Duration of TB treatment at cART initiation [median (IQR)]: 73 (44-115) days
Boulle 2010 (a,b)	Not specified
Chu 2011	Not specified
Dao 2011	Not specified
DeSilva 2009	Not specified
Dronda 2011	Duration of TB treatment at cART initiation [median (IQR)]: 53 (25.75-83.25) days
Greig 2012	Not specified
Gupta 2013	Not specified
Lartey 2011	Duration of TB treatment at cART initiation ranged from 4 to 90 days (median: 33)
Liechty 2007	Not specified
Makombe 2007 (a,b)	cART was deferred until the continuation phase of TB treatment (2 months) was complete.
Manosuthi 2010	Median (IQR) duration of concurrent administration of nevirapine and rifampin: 5.4 (4.6-6.1) months
Mugusi 2012 (a,b)	All patients diagnosed with TB started cART after 4 weeks of TB treatment.
Mutevedzi 2011 (a,b,c,d)	Not specified
Nguyen 2011	Not specified
Stringer 2006 (a,b)	Many patients were taking TB treatment for weeks to months prior to cART initiation.<br>If newly diagnosed with TB during enrollment, they deferred cART until the acute phase of TB treatment was complete, unless CD4 count <50 when cART was started immediately.
Westreich 2012 (a,b)	254 (21.2%) were on TB treatment for >120 days prior to cART initiation.<br>320 (26.7%) were on TB treatment for 61-120 days prior to cART initiation.<br>289 (24.1%) were on TB treatment for 31-60 days prior to cART initiation.<br>334 (27.9%) were on TB treatment for ≤30 days prior to cART initiation.
Zachariah 2006	Not specified
Zachariah 2009	Not specified

Abbreviations: cART, combination antiretroviral therapy; IQR, interquartile range; TB, tuberculosis
