# Supplementary material for: The Effect of Tuberculosis Treatment at Combination Antiretroviral Therapy Initiation on Subsequent Mortality: A Systematic Review and Meta-Analysis
Source: PLoS One. 2013 Oct 15;8(10):e78073. doi: 10.1371/journal.pone.0078073 (PMC3797056; doi:10.1371/journal.pone.0078073)
Supplement: Table S4 — Median (IQR) baseline CD4 cell count by TB treatment status, if available. (PDF) [file pone.0078073.s004.pdf]

Table S4. Median (IQR) baseline CD4 cell count by TB treatment status, if available

Study	TB treatment	No TB treatment
Bassett 2012	TB sputum culture positive at enrollment: 80 (41-125)<br>On TB treatment at enrollment: 65 (29-112)	104 (49-160)
Bera 2009	NA	NA
Bhowmik 2012	NA	NA
Boulle 2008 (a)	80 (42-137)	116 (58-167)
Boulle 2008 (b)	61 (27-117)	93 (37-155)
Boulle 2010 (a,b)	NA	NA
Chu 2011	NA	NA
Dao 2011	NA	NA
DeSilva 2009	NA	NA
Dronda 2011	80 (32-186)	226 (126-310)
Greig 2012	NA	NA
Gupta 2013	NA	NA
Lartey 2011	46	88
Liechty 2007	NA	NA
Makombe 2007 (a,b)	NA	NA
Manosuthi 2010	37 (17-77)	29 (8-112)
Mugusi 2012 (a,b)	94.5 (123)	90 (118)
Mutevedzi 2011 (a,b,c,d)	NA	NA
Nguyen 2011	NA	NA
Stringer 2006 (a,b)	NA	NA
Westreich 2012 (a,b)	58 (22-116)	94 (34-165)
Zachariah 2006	NA	NA
Zachariah 2009	NA	NA

Abbreviations: IQR, interquartile range; NA, not available; TB, tuberculosis
