# Supplementary material for: The Effect of Tuberculosis Treatment at Combination Antiretroviral Therapy Initiation on Subsequent Mortality: A Systematic Review and Meta-Analysis
Source: PLoS One. 2013 Oct 15;8(10):e78073. doi: 10.1371/journal.pone.0078073 (PMC3797056; doi:10.1371/journal.pone.0078073)
Supplement: Table S5 — Meta-regression results for the effect of TB treatment on mortality. (PDF) [file pone.0078073.s005.pdf]

Table S5. Meta-regression results for the effect of TB treatment on mortality

Length of follow-up time	Study or Population Characteristic	Category	Number of Studies	Homogeneity p-value	RR_{RE} (95% CI)	Ratio of RRs (95% CI)
1-3 months	Study design	Prospective cohort	5	0.13	1.11 (0.80, 1.54)	1.02 (0.49, 2.13)
		Retrospective cohort	3	0.10	1.08 (0.68, 1.71)	Reference
	Median baseline CD4 count	<100 cells/μL	2	0.08	1.97 (0.44, 8.84)	1.67 (0.40, 6.98)
		≥100 cells/μL	5	0.23	1.12 (0.89, 1.40)	Reference
	Ratio measure	Hazard ratio	4	0.42	1.19 (0.98, 1.44)	1.34 (0.71, 2.54)
		Risk ratio or odds ratio	4	0.07	1.02 (0.56, 1.86)	Reference
	Adjustment*	Adjusted	6	0.08	1.17 (0.89, 1.55)	1.41 (0.62, 3.22)
		Unadjusted	2	0.80	0.82 (0.52, 1.31)	Reference
	Adjustment for age and sex†	Yes	5	0.23	1.12 (0.89, 1.40)	1.04 (0.43, 2.49)
		No	3	0.05	1.32 (0.51, 3.40)	Reference
	Adjustment for hemoglobin	Yes	3	0.13	1.56 (0.99, 2.44)	1.55 (0.96, 2.49)
		No	5	0.64	0.96 (0.78, 1.18)	Reference
6-12 months	Study design	Prospective cohort	7	0.10	1.22 (0.86, 1.74)	1.06 (0.63, 1.79)
		Retrospective cohort	4	0.11	1.10 (0.85, 1.43)	Reference
	Length of follow-up	6-9 months	4	0.14	0.93 (0.68, 1.28)	0.70 (0.51, 0.96)
		11-12 months	7	0.61	1.29 (1.06, 1.56)	Reference
	Clinical setting	HIV care	7	0.10	1.19 (0.95, 1.49)	1.21 (0.68, 2.16)
		Primary care	4	0.11	1.13 (0.63, 2.03)	Reference
	cART regimen	EFV-based	3	0.62	0.86 (0.60, 1.23)	0.74 (0.40, 1.37)
		NVP-based or a mixture	8	0.03	1.23 (0.96, 1.58)	Reference
	TB is main exposure	Yes	6	0.25	0.99 (0.80, 1.23)	0.72 (0.44, 1.16)
		No	5	0.13	1.46 (1.00, 2.12)	Reference
	Gender	Women only	2	0.18	2.57 (0.83, 7.98)	2.08 (0.81, 5.33)
		Both genders included	9	0.16	1.08 (0.90, 1.29)	Reference
	Mean patient age	≤33 years	4	0.06	1.21 (0.68, 2.16)	0.80 (0.43, 1.48)
		>33 years	5	0.42	1.26 (1.03, 1.54)	Reference
	Median baseline CD4 count	<100 cells/μL	4	0.17	1.12 (0.75, 1.67)	0.84 (0.46, 1.52)
		≥100 cells/μL	6	0.15	1.33 (0.97, 1.83)	Reference
	Ratio measure	Hazard ratio	6	0.56	1.02 (0.84, 1.24)	0.79 (0.47, 1.33)
		Risk ratio	5	0.01	1.44 (0.91, 2.27)	Reference
	Adjustment‡	Adjusted	6	0.73	1.00 (0.82, 1.22)	0.75 (0.45, 1.26)
		Unadjusted	5	0.01	1.47 (0.95, 2.28)	Reference
	Adjustment for age and sex§	Yes	5	0.66	0.99 (0.81, 1.21)	0.73 (0.44, 1.22)
		No	6	0.01	1.46 (0.97, 2.20)	Reference
	Adjustment for BMI/weight	Yes	2	1.00	1.05 (0.80, 1.38)	0.87 (0.46, 1.68)
		No	9	0.03	1.21 (0.93, 1.59)	Reference
	Adjustment for hemoglobin	Yes	2	0.66	1.28 (0.74, 2.22)	1.11 (0.49, 2.53)
		No	9	0.03	1.15 (0.91, 1.45)	Reference
18-98 months	Study design	Prospective cohort	7	0.01	1.14 (0.91, 1.42)	0.54 (0.26, 1.13)
		Retrospective cohort	3	<0.01	2.21 (0.89, 5.49)	Reference
	Length of follow-up	18-36 months	3	<0.01	1.58 (0.81, 3.10)	1.24 (0.56, 2.76)
		47-98 months	7	<0.01	1.23 (0.94, 1.62)	Reference
	Geographic location	Africa	5	<0.01	1.24 (0.84, 1.82)	0.80 (0.37, 1.73)
		Asia or Europe	5	0.49	1.50 (1.25, 1.81)	Reference

Clinical setting	HIV care	8	<0.01	1.54 (1.12, 2.10)	1.70 (0.77, 3.76)
	Primary care	2	0.02	0.91 (0.57, 1.47)	Reference
All patients ART-naïve	Yes	8	<0.01	1.46 (0.99, 2.15)	1.30 (0.54, 3.14)
	No	2	0.75	1.12 (0.97, 1.28)	Reference
TB is main exposure	Yes	5	0.20	1.30 (1.08, 1.58)	0.92 (0.42, 2.01)
	No	5	<0.01	1.44 (0.85, 2.44)	Reference
Used national death registry	Yes	2	0.03	0.90 (0.58, 1.38)	0.57 (0.26, 1.24)
	No	8	<0.01	1.55 (1.14, 2.10)	Reference
Mean patient age	≤33 years	2	0.01	1.33 (0.33, 5.31)	0.84 (0.28, 2.54)
	>33 years	7	<0.01	1.41 (1.03, 1.93)	Reference
Median baseline CD4 count	<100 cells/μL	3	0.07	1.32 (0.92, 1.88)	1.05 (0.38, 2.94)
	≥100 cells/μL	5	<0.01	1.26 (0.76, 2.08)	Reference
Ratio measure	Hazard ratio or IRR	8	<0.01	1.35 (0.99, 1.85)	1.02 (0.36, 2.90)
	Risk ratio	2	0.88	1.38 (1.07, 1.77)	Reference
Adjustment**	Adjusted	8	<0.01	1.29 (0.99, 1.70)	0.63 (0.19, 2.12)
	Unadjusted	2	0.30	2.13 (0.96, 4.72)	Reference
Adjustment for age	Yes	7	<0.01	1.28 (0.93, 1.76)	0.77 (0.31, 1.92)
	No	3	0.32	1.51 (1.06, 2.14)	Reference
Adjustment for baseline hemoglobin	Yes	2	0.67	1.07 (0.90, 1.27)	0.71 (0.30, 1.70)
	No	8	<0.01	1.47 (1.04, 2.09)	Reference
Adjustment for BMI/weight	Yes	4	0.11	1.01 (0.84, 1.21)	0.53 (0.31, 0.90)
	No	6	<0.01	1.85 (1.25, 2.73)	Reference
Adjustment for baseline CD4 count	Yes	7	<0.01	1.32 (0.95, 1.85)	0.88 (0.35, 2.22)
	No	3	0.16	1.44 (0.82, 2.54)	Reference

Abbreviations: ART, antiretroviral therapy; BMI, body mass index; cART, combination antiretroviral therapy; CI, confidence interval; EFV, efavirenz; HIV, human immunodeficiency virus; IRR, incidence rate ratio; NVP, nevirapine; RR, relative risk; RRRE, random effects relative risk; TB, tuberculosis

* All of the adjusted estimates adjusted for baseline CD4 count and BMI or weight

† The 5 estimates that adjusted for age also adjusted for sex

‡ All of the adjusted estimates adjusted for baseline CD4 count

§ The 5 estimates that adjusted for age also adjusted for sex

** All of the adjusted estimates adjusted for sex
