# Supplementary material for: The Effect of Tuberculosis Treatment at Combination Antiretroviral Therapy Initiation on Subsequent Mortality: A Systematic Review and Meta-Analysis
Source: PLoS One. 2013 Oct 15;8(10):e78073. doi: 10.1371/journal.pone.0078073 (PMC3797056; doi:10.1371/journal.pone.0078073)
Supplement: Document S1 — Funnel plots of mortality relative risks and inverse-variance weights. (PDF) [file pone.0078073.s006.pdf]

Document S1. Funnel plots of mortality relative risks and inverse-variance weights

a)

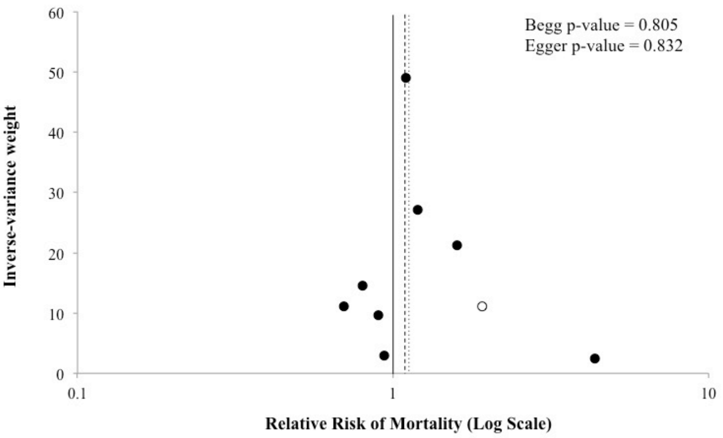

b)

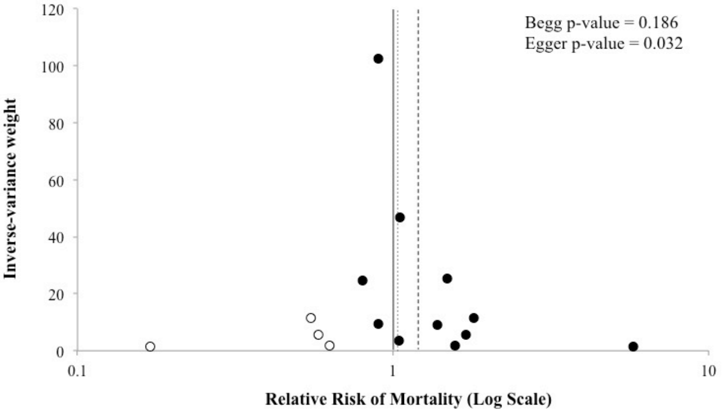

c)

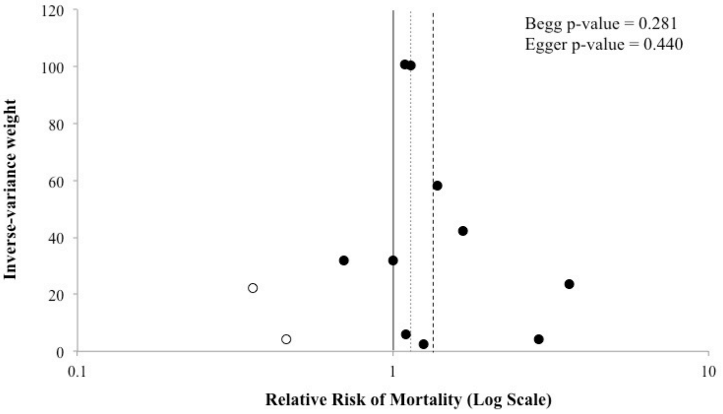

Funnel plots of mortality relative risks and inverse-variance weights at (a) 1-3 months, (b) 6-12 months, and (c) 18-98 months after combination antiretroviral therapy initiation. Black circles are reported results; white circles are imputed results from the trim-and-fill method. The solid line is the null value of 1. The dashed line represents the random effects summary relative risk. The dotted line represents the random effects summary relative risk with the imputed studies.
