# Supplementary material for: The Effect of Tuberculosis Treatment at Combination Antiretroviral Therapy Initiation on Subsequent Mortality: A Systematic Review and Meta-Analysis
Source: PLoS One. 2013 Oct 15;8(10):e78073. doi: 10.1371/journal.pone.0078073 (PMC3797056; doi:10.1371/journal.pone.0078073)
Supplement: Document S2 — Sensitivity analysis of asymmetrical confidence intervals. (PDF) [file pone.0078073.s007.pdf]

Document S2. Sensitivity analysis of asymmetrical confidence intervals

We conducted a three-part sensitivity analysis, in each part of which one of the three values would be replaced by a value imputed from the other two, assuming interval symmetry. With lnRR as the natural log of the point estimate, lnLO as the natural log of the lower limit and lnUP as the natural log of the upper limit:

a. imputed lnRR = 0.5(lnUP + lnLO)
b. imputed lnLO = lnRR – (lnUP – lnRR) = 2(lnRR) – lnUP
c. imputed lnUP = lnRR + (lnRR – lnLO) = 2(lnRR) – lnLO

We then re-ran the analysis with each calculated estimate and 95% CI to examine if and how the summarized random effects estimate and 95% CI change, for the appropriate time period.

Mutevedzi 2011a – 3-month estimate

**Iteration**	**Calculated estimate (95% CI)**	**Resulting summary effect estimate (95% CI)**
Asymmetrical interval:	1.59 (0.84, 1.97)	1.10 (0.87, 1.40)
a. imputed lnRR = 0.5[ln(1.97) + ln(0.84)] = 0.25	1.29 (0.84, 1.97)	1.07 (0.87, 1.32)
b. imputed lnLO = 2ln(1.59) – ln(1.97) = 0.25	1.59 (1.28, 1.97)	1.13 (0.87, 1.47)
c. imputed lnUP = 2ln(1.59) – ln(0.84) = 1.10	1.59 (0.84, 3.01)	1.07 (0.85, 1.36)

Boulle 2008b – 6-month estimate

**Iteration**	**Calculated estimate (95% CI)**	**Resulting summary effect estimate (95% CI)**
Asymmetrical interval:	0.8 (0.5, 1.1)	1.15 (0.94, 1.41)
a. imputed lnRR = 0.5[ln(1.1) + ln(0.5)] = -0.30	0.7 (0.5, 1.1)	1.14 (0.92, 1.42)
b. imputed lnLO = 2ln(0.8) – ln(1.1) = -0.54	0.8 (0.6, 1.1)	1.14 (0.93, 1.40)
c. imputed lnUP = 2ln(0.8) – ln(0.5) = 0.25	0.8 (0.5, 1.3)	1.16 (0.94, 1.42)

Though asymmetry is possibly due to rounding, not error.

Nguyen 2011 – 60-month estimate

**Iteration**	**Calculated estimate (95% CI)**	**Resulting summary effect estimate (95% CI)**
Asymmetrical interval:	2.9 (1.6, 10.5)	1.33 (1.02, 1.75)
a. imputed lnRR = 0.5[ln(10.5) + ln(1.6)] = 1.41	4.1 (1.6, 10.5)	1.37 (1.05, 1.79)
b. imputed lnLO = 2ln(2.9) – ln(10.5) = -0.22	2.9 (0.8, 10.5)	1.33 (1.02, 1.72)
c. imputed lnUP = 2ln(2.9) – ln(1.6) = 1.66	2.9 (1.6, 5.3)	1.38 (1.06, 1.80)
